# A systematic review and meta-analysis of the impact of left bundle branch area pacing on right ventricular function

**DOI:** 10.3389/fcvm.2025.1545757

**Published:** 2025-01-30

**Authors:** Lei Yin, Lianxia Wang, Jiankang Meng, Qian Liu, Yan Zhang, Yanlei Zhao, Mingwang Li, Ling You

**Affiliations:** Division of Cardiology, The Second Hospital of Hebei Medical University, Shijiazhuang, Hebei, China

**Keywords:** left bundle branch area pacing, right ventricular function, meta-analysis, right ventricular fractional area change, interventricular mechanical delay

## Abstract

**Objective:**

This study aims to systematically evaluate and perform a meta-analysis on the effects of LBBAP on right ventricular (RV) function by collecting data on Right Ventricular Fractional Area Change (RV-FAC), Tricuspid Annular Plane Systolic Excursion (TAPSE), Interventricular Mechanical Delay (IVMD), and the incidence of tricuspid regurgitation (TR) worsening in Left bundle branch area pacing (LBBAP) patients.

**Methods:**

A comprehensive search was conducted for studies published from the establishment of the respective databases until October 2024 in PubMed, Embase, Web of Science, and the Cochrane Library. After screening and data extraction, the Newcastle-Ottawa Scale was used for the quality assessment of the included cohort studies, and meta-analysis was performed using R software. The effect size was estimated using either a random-effect model or a fixed-effect model, with odds ratio (OR) and mean difference (MD).

**Results:**

A total of 14 studies were included, analyzing 1,555 LBBAP patients. The meta-analysis revealed that compared with intrinsic conduction, LBBAP implantation significantly improved RV-FAC (MD = 1.93; 95% CI: 0.64–3.23, *P* = 0.0034) and TAPSE (MD = 1.57; 95% CI: 1.07–2.06, *P* < 0.0001). Compared to the RVP group, LBBAP implantation significantly shortened IVMD (MD = −21.27; 95% CI: −31.33 to −11.22, *P* < 0.0001). For patients with RV dysfunction or right bundle branch block (RBBB), LBBAP implantation also significantly reduced IVMD (MD = −31.31; 95% CI: −37.10 to −25.52, *P* < 0.0001). The incidence of TR worsening within one year after LBBAP was approximately 8%, increasing to 23% beyond one year.

**Conclusion:**

This meta-analysis demonstrates the superiority of LBBAP over intrinsic conduction in improving RV systolic function. Compared to RVP, LBBAP significantly enhances biventricular synchronization. Furthermore, LBBAP also improves ventricular synchronization in patients with RV dysfunction or RBBB.

## 1 Introduction

As a traditional pacing modality for the treatment of bradyarrhythmias, right ventricular pacing (RVP) has been successfully used in clinical practice for several decades. However, in patients who are dependent on ventricular pacing, long-term RVP can lead to dyssynchronous ventricular activation and impaired left ventricular function, ultimately resulting in heart failure (1). His bundle pacing, which stimulates the intrinsic conduction system to pace the myocardium, offers significant clinical benefits for patients with bradyarrhythmias or heart failure. However, the application of His bundle pacing is still limited by several factors, such as the complexity of the procedure, higher pacing thresholds, and lower R-wave amplitudes (2).

In recent years, left bundle branch area pacing (LBBAP) has emerged as a novel approach to achieving physiological pacing. By capturing the left bundle branch either partially or fully, it achieves left ventricular activation comparable to that of His bundle pacing. Compared to His bundle pacing, LBBAP offers superior pacing characteristics, including lower pacing thresholds and higher R-wave amplitudes (3). Due to these advantages, LBBAP has been widely adopted in clinical practice.

Despite the numerous advantages of left bundle branch area pacing (LBBAP), it also has certain limitations. While LBBAP achieves rapid left ventricular activation, it often results in delayed activation of the right ventricle, with the pacing electrocardiogram typically showing a right bundle branch block (RBBB) pattern (4). Although RBBB is generally considered benign, some clinical studies have suggested that patients with complete right bundle branch block and concomitant cardiopulmonary disease tend to have a higher incidence of adverse events (5, 6). Unlike the left heart system, clinical studies on the effects of LBBAP on the right heart system are primarily based on small cohort studies, and large-scale data comparisons are lacking. Therefore, a systematic review of the impact of LBBAP on right ventricular (RV) function is particularly important.

The article performs a systematic review and meta-analysis by collecting data on right ventricle fractional area change (RV-FAC), tricuspid annular systolic excursion (TAPSE), interventricular mechanical delay (IVMD), and the rate of tricuspid regurgitation (TR) deterioration in LBBAP patients, with the aim of investigating the impact of LBBAP on RV function.

## 2 Methods

### 2.1 Data sources and searches

We systematically searched the PubMed, Cochrane Library, Web of Science, and Embase databases for studies published from their inception through October 14, 2024, focusing on the impact of LBBAP on RV function. The search strategy for the databases, using PubMed as an example, was as follows: [left bundle branch pacing(Title/Abstract)] OR [left bundle branch area pacing(Title/Abstract)]. This study synthesized data from previously published research, and therefore, ethical approval was not required.

### 2.2 Study design

This study followed the Preferred Reporting Items for Systematic Reviews and Meta-Analyses (PRISMA) guidelines for reporting systematic reviews and meta-analyses (7). Clinical studies were considered eligible for inclusion if they met the following criteria: (1) Randomized controlled trials, cohort studies, case-control studies, or single-arm studies. (2) The study investigated the impact of LBBAP on RV function (non-acute hemodynamic changes). (3) Full-text articles published in English in peer-reviewed journals. (4) For studies with multiple publications from the same research, only the study with the largest population was included. Studies without original data, reviews, case reports, editorials, and animal studies were excluded.

### 2.3 Data extraction and quality appraisal

Two independent reviewers (Lei Yin and Lianxia Wang) conducted the search and reviewed the titles, abstracts, and full texts to determine eligible studies. Any disagreements regarding the decisions were resolved through consultation with a third-party reviewer (Ling You). The data extracted primarily included: (1) Baseline information: authors, publication year, study design, number of patients, and follow-up duration. (2) Primary clinical outcome measures: RV-FAC%, TAPSE. Secondary outcome measures: IVMD, TR deterioration rate. The quality of the included studies was assessed using the Newcastle-Ottawa Scale for cohort studies.

### 2.4 Statistical analysis

Data were analyzed using R version 4.2.1 statistical software (https://www.r-project.org, The R Foundation). The primary outcomes analyzed were RV-FAC, TAPSE, IVMD, and the TR deterioration rate. Descriptive statistics for continuous variables were expressed as means and standard deviations (SD), while categorical variables were presented as counts (n) and percentages (%). Values expressed as medians (interquartile range, IQR) were converted to means ± standard deviation for statistical analysis. For continuous variables, the difference in means (MD) with 95% confidence intervals (CI) was used for statistical description. For categorical variables, odds ratios (OR) with 95% CI were used for statistical description. Heterogeneity across studies was assessed using the *I*^2^ statistic. If *I*^2^ > 50%, significant heterogeneity was assumed, and a random-effects model was applied; otherwise, a fixed-effects model was used. In the presence of significant heterogeneity, sensitivity analyses were performed by sequentially omitting one study at a time to assess the impact of each individual study on the overall risk estimate. Potential publication bias was evaluated using Egger's test. Statistical significance was set at *p* < 0.05.

## 3 Results

### 3.1 Study selection and quality assessment

**Figure 1:**
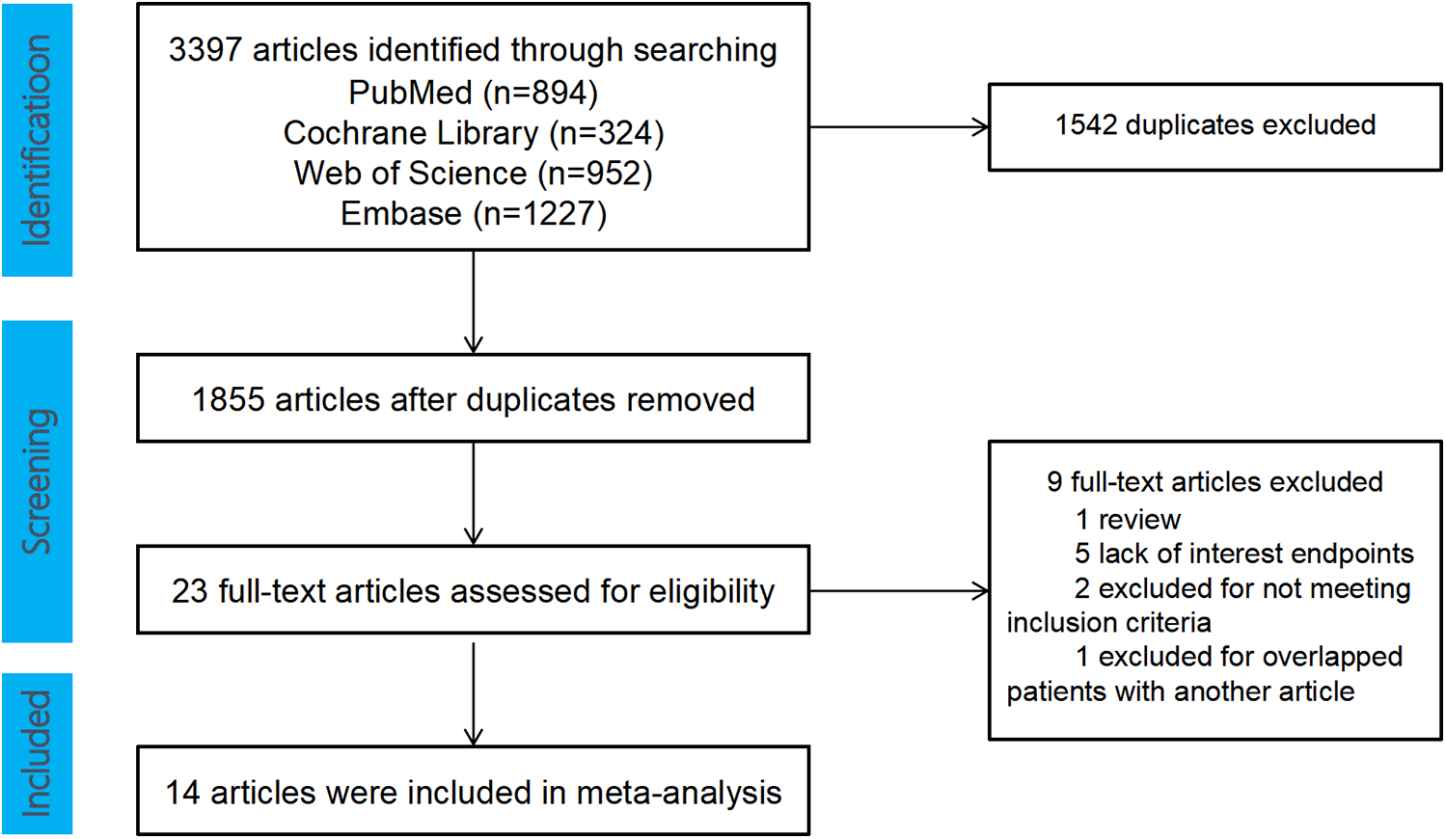
Flow diagram of the study selection.

A total of 3,397 articles were retrieved from the following databases: PubMed (894), Cochrane Library (324), Web of Science (952), and Embase (1,227). After removing duplicates, 1,855 articles remained. These articles were screened by reviewing the titles, abstracts, and full texts, resulting in 23 clinical studies for further evaluation (see Figure 1). Among these, two studies originated from the same center, with patient enrollment occurring from January 2018 to August 2020. Since some patients were overlapping in these studies, we ultimately selected the study with the largest sample size (8, 9). Additionally, two clinical studies, which focused on acute-phase right ventricular hemodynamic parameters, were excluded from the analysis (10, 11). In the end, 14 clinical studies met our inclusion criteria. All the included studies were cohort studies, comprising a total of 1,555 LBBAP patients. The demographic and clinical characteristics of the included studies are presented in Table 1.

**Table 1:** Study baseline characteristics of patients included in the analysis.

Authors	Year	Regions	Study patients	Interventions	Follow-up (months)	Outcomes	NOS
Mao et al. (12)	2023	China	SSS in 35.9% of patients, AVB in 37.2% and atrial fibrillation with slow heart rate in 11.5%.	45 LBBP vs. 33 RVP	12	④	9
Sun et al. (13)	2020	China	III AVB or II AVB sick sinus syndrome with ventricular pacing ratio >70%;	16 LBBP vs. 16 RVP	NA	③	7
Liu et al. (14)	2022	China	According to 2013 ESC/EHRA Guidelines	33 LBBP vs. 21 RVP	13.94 ± 4.44 vs. 13.56 ± 4.64	③	9
Huang et al. (15)	2022	China	SSS with normal cardiac function	42 LBBP vs. 42 RVP	NA	①③	7
Yang et al. (16)	2024	China	AVB	16 LBBAP vs. 13 RVP	40 vs. 30	③	9
Liu et al. (17)	2021	China	(a) Patients with LVEF ≤35% despite optimal medical treatment for at least 3 months; (b) CLBBB morphology and QRSd ≥ 130 ms; and (c) age ≥18 years old.	27 LBBAP vs. 35 BVP	3	②③	8
Li et al. (18)	2020	China	Complete LBBB and RBBB	LBBAP(LBBB *N* = 19; RBBB *N* = 27)	6	①②	8
Mirolo et al. (19)	2022	France	The decision of LBBAP rather than conventional pacing was left to operators’ discretion.	134 LBBAP(RBBB *N* = 33)	12	③	8
Tian et al. (20)	2023	China	Inclusion criteria were age >18 years who had indications for CRT	LBBAP(No RV dysfunction *N* = 30; RV dysfunction *N* = 35)	6	①②③④	8
Bednarek et al. (21)	2024	Poland	Any indication for permanent pacing due to bradycardia	122 LBBAP（LBBP *N* = 109; LVSP *N* = 13 ）	21	①②④	9
Mao et al. (22)	2024	China	Patients had symptomatic bradycardia	31 LBBP vs. 29 RVP	12	④	9
Hu et al. (8)	2022	China	According to AHA/ACC/HRS Guidelines	91 LBBAP	20.5 (17.0, 24.0) vs. 15.0 (12.5, 21.5)	④	9
Su et al. (3)	2021	China	With an indication for treatment of bradycardia or cardiac resynchronization therapy.	618 LBBAP	12	④	9
Li et al. (23)	2022	China	According to AHA/ACC/HRS Guidelines	269 LBBAP vs. 203 RVP	24	④	9

SSS, sick sinus syndrome; AVB, atrioventricular block; LBBP, left bundle branch pacing; RVP, right ventricular pacing; LBBAP, left bundle branch area pacing; LVSP, left ventricular septum pacing; ①, right ventricle fractional area change (RV-FAC); ②, tricuspid annular plane systolic excusion (TAPSE); ③, interventricular Mechanical Delay (IVMD); ④, the tricuspid regurgitation deterioration rate.

### 3.2 Effect of LBBAP on right ventricular fractional area change (RV-FAC)

**Figure 2:**
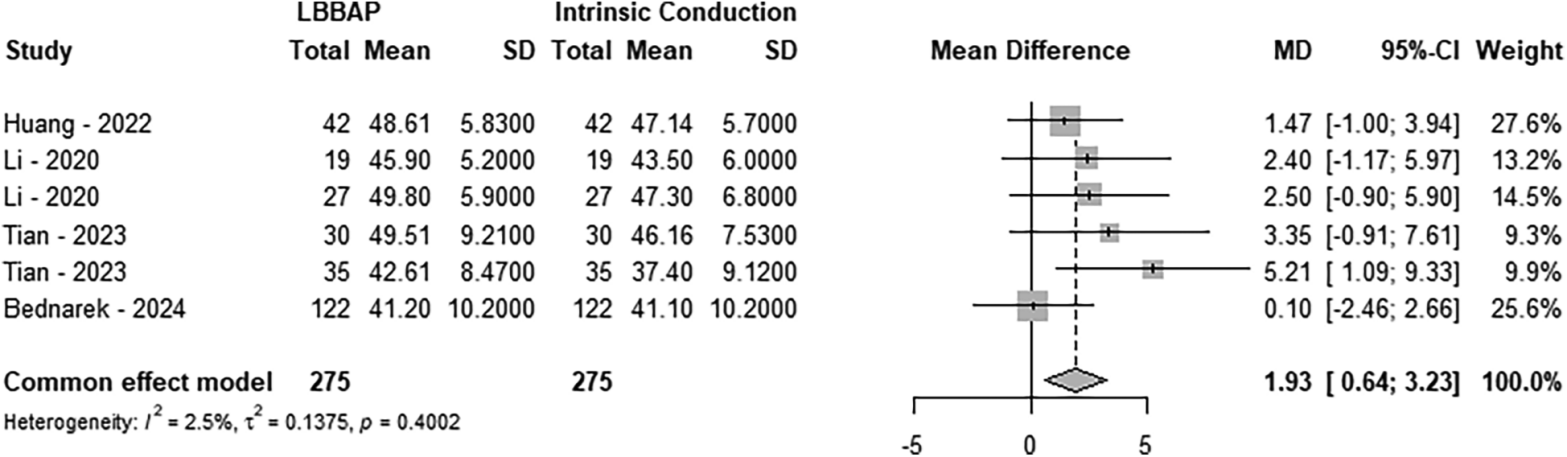
Forest plot of the right ventricle fractional area change. Comparison of the right ventricle fractional area change between LBBAP and intrinsic conduction.

A total of four studies were included, involving 275 LBBAP patients. The analysis showed no significant heterogeneity between the two groups (*P* = 0.4002, *I*^2^ = 2.5%), so a fixed-effects model was used. Sensitivity analysis revealed that the combined interval did not show significant variation, ranging from 1.5759 [0.2119; 2.9400] to 2.5655 [1.0641; 4.0668]. Although the Egger test indicated the presence of publication bias (*P* = 0.0271), the trim-and-fill method did not reveal any significant changes in the results, suggesting the findings were robust (MD = 1.37; 95% CI: 0.19–2.56, *P* = 0.0233). When compared with intrinsic conduction, the implantation of LBBAP significantly improved RV-FAC in patients (MD = 1.93; 95% CI: 0.64–3.23, *P* = 0.0034) (see Figure 2).

### 3.3 Impact of LBBAP on tricuspid annular plane systolic excursion (TAPSE)

**Figure 3:**
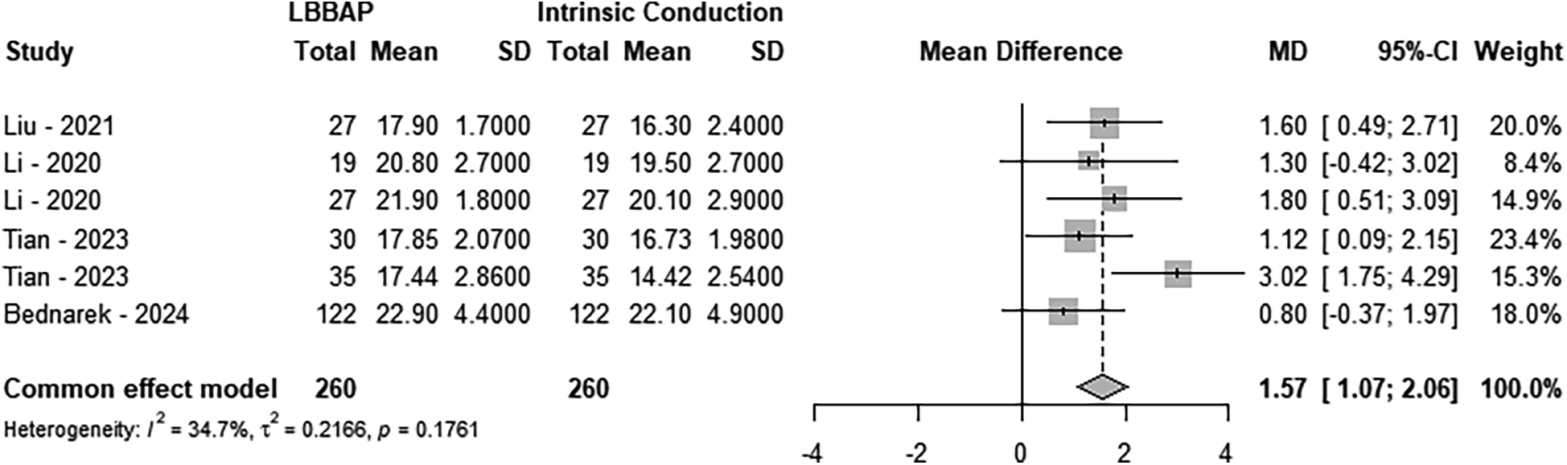
Forest plot of the tricuspid annular plane systolic excusion. Comparison of the tricuspid annular plane systolic excusion between LBBAP and intrinsic conduction.

A total of four studies, comprising 260 LBBAP patients, were included in the analysis. The results indicated no significant heterogeneity between the two groups (*P* = 0.1761, *I*^2^ = 34.7%), and a fixed-effect model was applied. Sensitivity analysis demonstrated that the combined interval did not exhibit significant variation, with a range from 1.3023 [0.7631; 1.8415] to 1.7340 [1.1860; 2.2821]. Furthermore, the Egger test revealed no publication bias (*P* = 0.67). Compared to intrinsic conduction, LBBAP implantation significantly improved patients’ TAPSE (MD = 1.57; 95% CI: 1.07–2.06, *P* < 0.0001), as shown in Figure 3.

### 3.4 Impact of LBBAP on interventricular mechanical delay (IVMD)

**Figure 4:**
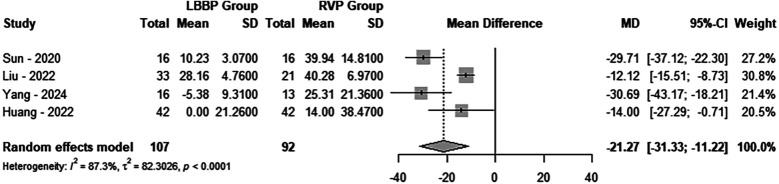
Forest plot of the interventricular mechanical delay. Comparison of the interventricular mechanical delay between LBBAP group and RVP group.

A total of 4 studies involving 199 patients were included in this analysis, with 107 patients in the LBBAP group and 92 patients in the RVP group. The results revealed significant heterogeneity between the two groups (*P* < 0.0001, *I*^2^ = 87.3%). Consequently, a random-effects model was applied. Sensitivity analysis demonstrated no substantial variation in the combined confidence intervals, which ranged from −18.0179 [−29.2156; −6.8202] to −25.6888 [−35.2327; −16.1450]. Furthermore, the Egger test indicated no evidence of publication bias (*P* = 0.3180). When comparing the two groups, LBBAP implantation significantly reduced IVMD compared to RVP (MD = −21.27; 95% CI: −31.33 to −11.22, *P* < 0.0001) (Figure 4).

**Figure 5:**
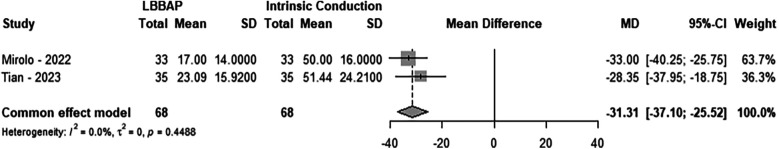
Forest plot of the interventricular mechanical delay. Comparison of the interventricular mechanical delay between LBBAP group and intrinsic conduction.

To further investigate the impact of LBBAP on RV function, we included two studies focused on patients with RV dysfunction or RBBB, comprising a total of 68 patients who received LBBAP. The results showed no significant heterogeneity between the two groups (*P* = 0.4488, *I*^2^ = 0.0%), allowing for the use of a fixed-effects model. Compared to native conduction, LBBAP implantation significantly reduced the IVMD in patients with RV dysfunction or RBBB (MD = −31.31; 95% CI: −37.10 to −25.52, *P* < 0.0001) (Figure 5).

### 3.5 Incidence of tricuspid regurgitation worsening in LBBAP patients

**Figure 6:**
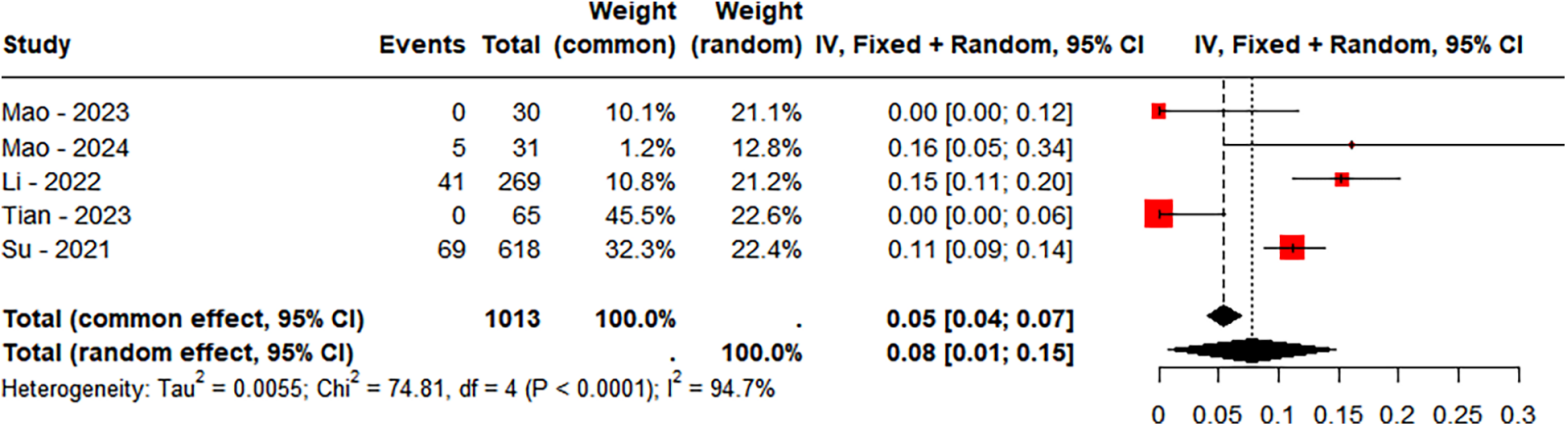
Incidence of tricuspid regurgitation (TR) deterioration (follow-up length ≤12 months).

A total of 7 studies were included, comprising 1,226 LBBAP patients. Based on the follow-up duration, the studies were divided into two groups. In the group with a follow-up duration ≤12 months, 5 studies were included, totaling 1,013 LBBAP patients. The incidence of TR worsening was found to follow a normal distribution and was analyzed using the raw rate for meta-analysis (Shapiro-Wilk normality test = 0.6923). The results showed significant heterogeneity between the two groups (*P* < 0.0001, *I*^2^ = 94.7%), and a random-effects model was applied. Sensitivity analysis demonstrated no significant changes in the combined interval, ranging from 0.06 [0.0000; 0.1294] to 0.10 [0.0258; 0.1745]. Moreover, Egger's test revealed no publication bias (*P* = 0.5558). In the group with follow-up ≤12 months, 8% of the population experienced worsening of TR (Figure 6).

**Figure 7:**
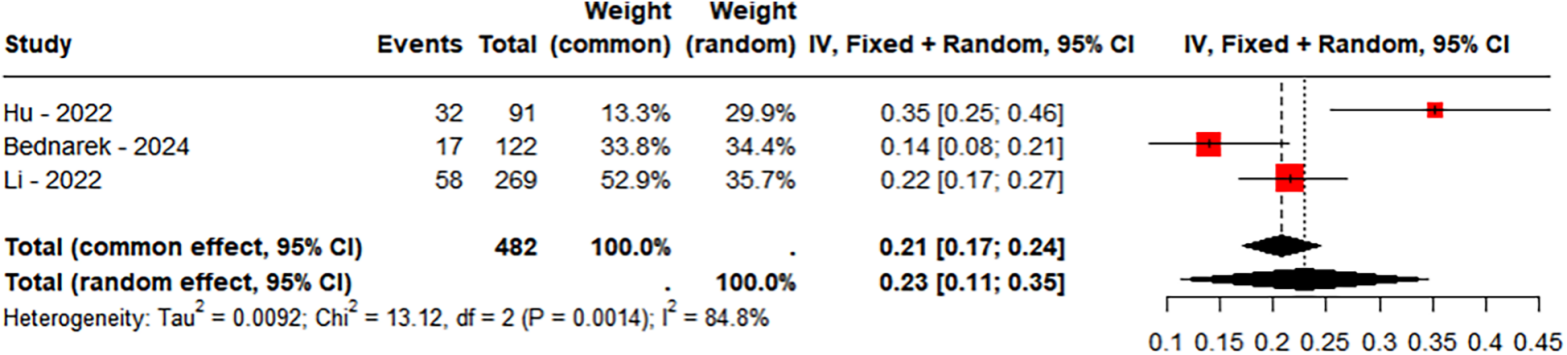
Incidence of tricuspid regurgitation (TR) deterioration (follow-up length >12 months).

A total of 3 studies were included, comprising 482 LBBAP patients, in the group with follow-up duration >12 months. The incidence of TR worsening followed a normal distribution and was analyzed using the raw rate for meta-analysis (Shapiro–Wilk normality test = 0.6923). The results revealed significant heterogeneity between the two groups (*P* = 0.0014, *I*^2^ = 84.8%), and a random-effects model was applied. Sensitivity analysis showed that the combined interval ranged from 0.1798 [0.1052; 0.2544] to 0.2767 [0.1441; 0.4094]. Furthermore, Egger's test indicated no evidence of publication bias (*P* = 0.5788). In the group with follow-up >12 months, 23% of patients experienced worsening of TR (Figure 7).

## 4 Discussion

The main findings of this study are as follows:

1. LBBAP significantly improves right ventricular systolic function in patients with pacing indications.
2. Compared to RVP, LBBAP significantly improves left and right ventricular synchrony. Additionally, LBBAP also enhances interventricular synchrony in patients with right ventricular dysfunction or right bundle branch block.
3. Meta-analysis results indicate that the incidence of tricuspid regurgitation worsening within the first year after LBBAP is approximately 8%, whereas the TR worsening rate increases to 23% after one year.

### 4.1 Right ventricular systolic function in LBBAP patients

Although LBBAP patients often present with RBBB patterns on their electrocardiogram (ECG), this does not appear to significantly impact RV systolic function. In the current study, RV-FAC and TAPSE were used to assess post-operative RV systolic function in LBBAP patients. RV-FAC typically reflects overall RV systolic function, while TAPSE assesses longitudinal RV systolic function. The results showed that both RV-FAC and TAPSE were significantly improved in LBBAP patients compared to those with intrinsic conduction. These findings are generally consistent with previous clinical studies (12–14). Additionally, Bednarek et al. (14) conducted a follow-up study of 122 LBBAP patients, with a median duration of 21 months. Through multivariate analysis, they identified that the improvement in left ventricular ejection fraction was the main independent predictor of improved RV systolic function in LBBAP patients. The interval or amplitude of the terminal R wave in lead V1 showed no statistical significance in relation to RV systolic function improvement, suggesting that the RBBB pattern in LBBAP patients may differ from conventional RBBB. Previous studies have also indicated that 20%–40% of RV systolic pressure and flow are influenced by left ventricular contraction (15). Considering that LBBAP significantly improves left ventricular electromechanical synchrony and reduces mitral regurgitation (16, 17), the interaction between the left and right ventricles may be a key mechanism driving the observed improvement in RV function following LBBAP. Furthermore, LBBAP may improve RV function by preferentially activating the left ventricle (13).

### 4.2 Synchronous function of the left and right ventricles

Ozpak et al. evaluated right ventricular activation by measuring the time from the QRS onset to the peak of the R wave in lead V1 (V1RWPT). They found that the V1RWPT in LBBAP patients was between that of complete RBBB and incomplete RBBB, with a tendency toward incomplete RBBB [42 ms (28, 55) vs. 42 ms (35, 49), *P* = 0.49] (18). This suggests that the RBBB induced by LBBAP may be improved, or even corrected, through mechanisms such as lateral connections between the left and right bundle branches, retrograde conduction of the left bundle branch signal, and intermyocardial electrical conduction (4). Our meta-analysis results also demonstrate that LBBAP patients, compared to those undergoing RVP, have a significantly shorter IVMD. Additionally, the findings suggest that LBBAP improves interventricular synchrony in patients with RV dysfunction or RBBB. Some studies also examined the effects of LBBAP in both non-RBBB and RBBB patients. The results revealed no significant differences between the two groups in right ventricular free wall strain, the standard deviation of peak time across three segments of the right ventricular free wall, TAPSE, or IVMD. However, in the RBBB group, there was a significant delay in the peak time difference during systole between the tricuspid and mitral valves (△TSTV-MV) and the displacement peak time difference (△PTTV-MV) when compared to the non-RBBB group (47.29 ± 58.45 vs. −12.00 ± 49.91, *P* = 0.02; 28.14 ± 39.04 vs. −28.00 ± 48.26, *P* = 0.01) (19). These findings suggest that LBBAP may be a viable option for patients with right bundle branch block who require pacing intervention.

In addition, recently proposed techniques such as anodal capture and bilateral bundle capture have provided alternative feasible options for patients with right bundle branch block. Ali et al (20). demonstrated that anodal capture significantly shortened both the QRS duration and total ventricular activation time (116 ± 12 ms vs. 129 ± 14 ms, *P* < 0.01 and 83 ± 18 ms vs. 90 ± 15 ms, *P* = 0.01). Lin et al. (21) achieved favorable pacing electrocardiographic parameters by simultaneously capturing both the left and right bundle branches using the tip and ring electrodes of the lead. As implantation techniques mature and lead technology improves, these approaches may have broader clinical applications in the future (22).

### 4.3 Incidence of tricuspid regurgitation (TR) deterioration

Due to the implantation of the LBBAP electrode in the right ventricular septum, improper handling during the procedure may potentially damage surrounding structures, thereby affecting RV function. Our meta-analysis results indicate that in the population with a follow-up time of ≤12 months, approximately 8% of LBBAP patients experience deterioration of tricuspid regurgitation. In the population with a follow-up time of >12 months, the incidence of TR deterioration in LBBAP patients rises to 23%. The comparison of TR deterioration rates between the RVP and LBBAP groups is currently a topic of interest. However, given that the meta-analysis results for the RVP and LBBAP groups in the included studies were not robust, we did not perform further analysis. Although the original research team of LBBAP suggested that approximately 30% of LBBAP patients experience improvement in TR, it remains critical to avoid damage to tricuspid valve structures during the surgical procedure (3). A recent meta-analysis identified the distance between the lead-implant site and the tricuspid valve annulus (lead-TA distance) as an important predictor of TR deterioration. A longer lead-TA distance significantly reduces the likelihood of TR deterioration (23). Two related studies from Fuwai Hospital, which used intraoperative mapping of the left bundle branch potential, suggested that the left bundle branch trunk is primarily located 15–35 mm from the tricuspid valve annulus. Based on these findings, they recommend that the LBBAP electrode be implanted at a distance greater than 19 mm from the tricuspid valve annulus to reduce the risk of TR deterioration (24, 25). In addition, the use of intracardiac echocardiogram during the procedure can further reduce the occurrence of such complications (26).

This study has several limitations: ① All the studies included in this research are cohort studies, and the lack of randomized controlled trials means that the findings are not corroborated by more rigorous study designs. ② The definition of left bundle branch pacing in the existing literature is inconsistent, and we did not clearly distinguish between left bundle branch pacing and left ventricular septal pacing. This lack of differentiation may have impacted the accuracy of our results to some extent. ③ The literature on evaluating right heart function in LBBAP patients is relatively limited, and the methods used are somewhat homogeneous. As a result, this study was unable to provide a comprehensive and detailed assessment of the effects of LBBAP on the right heart system. Research on Doppler tissue and strain assessment will provide more information on RV function. ④ Given that LBBAP may influence right heart function through improvement in left ventricular function, the improvement in RV function might be more significant in populations with reduced ejection fraction. However, due to limitations in the included studies, we were unable to conduct further subgroup analyses based on ejection fraction. ⑤ The studies included in this research all used the Medtronic 3,830 lead, and did not further explore the impact of Stylet-driven leads on right ventricular function.

## 5 Conclusion

This meta-analysis demonstrates the superiority of LBBAP over intrinsic conduction in improving RV systolic function. Compared to RVP, LBBAP significantly enhances biventricular synchronization. Furthermore, LBBAP also improves ventricular synchronization in patients with RV dysfunction or right bundle branch block (RBBB).
